# Evaluation of the physicochemical properties and adhesive interface quality of BioRoot Flow and BioRoot RCS endodontic sealers: An in vitro study

**DOI:** 10.1007/s00784-026-07084-3

**Published:** 2026-09-02

**Authors:** Dione Tavares Reis, Gustavo Alexandre de Castro-Vasconcelos, Gustavo Creazzo, Helena Cristina de Assis, Igor Bassi Ferreira Petean, Manoel Damiao de Sousa-Neto, Jardel Francisco Mazzi-Chaves, Fabiane Carneiro Lopes-Olhê

**Affiliations:** Department of Restorative Dentistry, School of Dentistry of Ribeirão Preto, University of São Paulo (USP), Av. do Café, s/n., Ribeirão Preto, SP 14020-904 Brazil

**Keywords:** Bioceramic sealer, Physicochemical properties, Bond strength

## Abstract

**Objective:**

Bioceramic sealers have been increasingly used in endodontics due to their favorable physicochemical and biological properties. However, evidence regarding the physicochemical properties, push-out bond strength, and adhesive interface characteristics of BioRoot Flow compared with other root canal sealers remains limited. Therefore, this study evaluated the physicochemical properties, push-out bond strength, failure pattern, and adhesive interface quality of BioRoot Flow (BRF), BioRoot RCS (BRR), and the epoxy resin–based sealer AH Plus (AHP).

**Methods:**

Setting time, dimensional change, solubility, radiopacity, and flow were assessed following ANSI/ADA Specification No. 57. Setting time was recorded using cylindrical molds tested with a Gillmore needle. Dimensional change was measured in five specimens per sealer before and after 30 days in distilled water. Solubility was assessed in ten specimens after seven days of immersion, followed by desiccation. Radiopacity was evaluated using acrylic plates radiographed beside an aluminum step wedge. Flow was assessed according to ANSI/ADA standards. For bond strength and interface analysis, thirty single-rooted teeth were prepared with the Reciproc system and irrigated with sodium hypochlorite and EDTA. Canals were obturated with the single-cone technique using the tested sealers (*n* = 10). Three slices were obtained from each root third; two were used for push-out testing and failure analysis, and one for confocal laser scanning microscopy.

**Results:**

AHP showed the shortest setting time, highest radiopacity, and greatest flow. BRF exhibited the highest solubility, while dimensional change did not differ among materials. Bond strength varied significantly among sealers and root thirds; AHP presented the highest values, followed by BRF and BRR. Adhesive failures predominated for AHP, whereas bioceramic sealers showed mainly mixed failures.

**Conclusion:**

AHP demonstrated superior physicochemical properties, higher bond strength, and better adhesive interface adaptation. BRF performed better than BRR regarding bonding and interface quality.

## Introduction

The objective of root canal system obturation is to achieve a three-dimensional filling using gutta-percha cones and endodontic sealers [1, 2]. The sealers should fill the spaces between the gutta-percha cones and the dentinal walls, reaching not only the main canal but also the accessory and lateral canals, as well as the irregularities of the root canal system. This ensures a three-dimensional seal capable of preventing microbial infiltration [3, 4].

Thus, the success of the obturation stage is directly related to the characteristics and physicochemical properties of the root canal sealer [5, 6]. Therefore, the sealer should exhibit low solubility (≤ 3%), dimensional stability, good flow, adequate setting time, and proper radiopacity [7], as well as antimicrobial activity and biocompatibility [3, 6, 8–10]. New endodontic sealers are constantly being introduced to the market in order to improve the quality of endodontic treatments, with bioceramic sealers standing out due to their calcium silicate composition and their remarkable biocompatibility and favorable biological properties, such as lower cytotoxicity [11] and superior antimicrobial activity compared to resin-based sealers [8, 10, 12]. Consequently, these sealers hold great clinical value, as they promote periapical healing [13] and have the potential to increase root strength after obturation [14].

The epoxy resin–based AH Plus sealer (Dentsply De Trey GmbH, Konstanz, Germany) is considered the gold standard among endodontic sealers and has been extensively evaluated regarding its physicochemical properties, biological response, and the adaptation of the adhesive interface formed with the intracanal dentin [6, 9, 14, 15]. This is due to its low solubility [15], high flow, excellent apical sealing ability [15], acceptable biocompatibility, low toxicity, and ability to form resin tags [15, 16], which favor the interlocking of the material [17–19] and consequently enhance adhesion to root dentin. Thus, this sealer provides higher bond strength to root dentin [19–22] and reduces marginal microleakage [23], in addition to exhibiting antimicrobial activity and desirable biological properties [8, 24, 25]. Although gutta-percha associated with resin-based sealers such as AH Plus remains the most commonly used material in obturation procedures [17, 18, 20], numerous techniques and materials with distinct physicochemical and biological properties—such as bioceramic sealers—have been developed [17, 20].

Among bioceramic endodontic sealers, BioRoot RCS (Septodont, Saint-Maur-des-Fossés, France) stands out as a powder–liquid system. Studies have shown that BioRoot RCS provides higher bond strength values [4] and a smaller volume of residual material after the retreatment of root canals obturated with this sealer compared to resin-based sealers [26]. The literature also reports that BioRoot RCS exhibits better adhesive interface adaptation when compared to another type of bioceramic sealer [27], as well as reduced postoperative pain and improved periapical healing, reinforcing its position as a suitable choice for endodontic practice [8, 26, 28].

Recently, BioRoot Flow (Septodont, Saint-Maur-des-Fossés, France) was introduced to the market. It is presented as a premixed paste containing highly pure calcium silicate mineral. According to the manufacturer, this composition prevents material shrinkage, thereby reducing the formation of gaps and voids during obturation, while also improving the material’s flowability. Furthermore, the manufacturer states that this sealer has an alkaline pH, which contributes to creating an environment unfavorable to bacterial proliferation, in addition to exhibiting good radiopacity. These characteristics position BioRoot Flow as a promising choice among endodontic sealers, standing out for its effectiveness and convenience. However, to date, no studies have been published evaluating the properties of this new material.

The growing popularity of bioactive sealers is attributed to their clinical benefits and advantageous biological properties, such as antimicrobial activity resulting from the promotion of an alkaline pH, as well as biocompatibility and bioactivity [8, 28–33]. In this context, premixed, ready-to-use bioceramic endodontic sealers have gained commercial prominence due to their ease of use. However, the setting reaction of these materials depends on the presence of moisture [29, 34]. Therefore, given the lack of studies on the newly introduced BioRoot Flow and considering that it is a premixed hydraulic sealer, further research is needed to evaluate its physicochemical properties and compare its bond strength to that of the powder–liquid BioRoot RCS, thereby assessing its clinical applicability.

## Objective

The present study aims to evaluate the physicochemical properties, bond strength, failure pattern, and quality of the adhesive interface after obturation with BioRoot Flow and BioRoot RCS sealers.

*Specific Objectives*.

1. To evaluate the setting time, dimensional change, solubility, radiopacity, and flow;
2. To assess, through a push-out test, the bond strength of the root canal sealers to dentin;
3. To classify, using a stereomicroscope, the failure pattern observed after the push-out test;
4. To evaluate, by means of confocal laser scanning microscopy, the quality of the formed adhesive interface.

## Materials and methods

The manuscript of this laboratory study has been written according to Preferred Reporting Items for Laboratory studies in Endodontology (PRILE) 2021 guidelines. To evaluate the physicochemical properties—setting time, flow, dimensional change, solubility, and radiopacity—of the bioactive endodontic sealers BioRoot Flow and BioRoot RCS (Septodont, Saint-Maur-des-Fossés, France), tests were conducted in accordance with ANSI/ADA Specification No. 57 (2021) [35], as modified by ISO 6876:2012, for root canal sealing materials that require moisture. For this purpose, the tested sealers were stored at 23 ± 2 °C and 95 ± 5% relative humidity for 24 h prior to testing. The components of the sealers used in this study are presented in Table 1. The materials were handled according to the manufacturer’s instructions. For the physicochemical tests (setting time, flow, dimensional change, solubility and radiopacity), BioRoot Flow and BioRoot RCS were manipulated with the incorporation of 1% distilled water by weight, according to the ANSI/ADA specification (ANSI/ADA, 2021), since these hydraulic calcium silicate-based sealers require moisture for setting.

**Table 1:** Characteristics of BioRoot Flow, BioRoot RCS, and AH Plus sealers

Material Composition	Manufacture	Apresentation
BioRoot Septodont Ready-to-use paste	Flow	Tricalcium silicate, propylene glycol, povidone, calcium carbonate, AEROSIL, zirconium oxide, sodium acrylate/acryloyldimethyltaurate copolymer, isohexadecane, and polysorbate.
BioRoot Septodont Powder/liquid	RCS	**Powder:** Tricalcium silicate, zirconium oxide, povidone. **Liquid:** Dihydrated calcium chloride, aero, purified water
AH Plus Dentsplay Paste/Paste with automixing system	Sirona	**Paste A:** Bisphenol-A epoxy resin, Bisphenol-F epoxy resin, calcium tungstate, zirconium oxide, silica, and iron oxide pigments. **Paste B:** Dibenzyldiamine, aminoadamantane, tricyclodecanediamine, calcium tungstate, zirconium oxide, silica, and silicone oil.

### Setting time

For the setting time test, five stainless steel circular molds measuring 5 mm in diameter and 2 mm in thickness were fixed onto a glass plate using utility wax. After mixing the sealer according to the manufacturer’s instructions, it was placed into the molds until completely filled. After 150 s, a 100 g Gillmore-type needle with an active tip of 2 mm in diameter was vertically positioned on the surface of the material at 60-second intervals. The procedure was repeated until no indentation was observed on the material’s surface, ensuring that the needle tip was cleaned after each measurement.

The setting time was defined as the period from the start of mixing until the moment when the sealer surface was no longer indented by the needle. The final setting time was determined as the arithmetic mean of the five test repetitions for each storage condition evaluated.

### Flow

For the flow test, a graduated syringe was used and filled with 0.5 mL of each sealer, mixed according to the manufacturer’s instructions. After 180 s, the material was carefully dispensed onto the center of a glass plate measuring 10 × 10 cm. Subsequently, a second glass plate was placed over the sealer, along with an additional weight, so that the total weight of the plate and load was 120 g. After 10 min, the largest and smallest diameters of the resulting disc were measured using a digital caliper.

For the collected measurements to be considered valid, the difference between the minimum and maximum diameters should not exceed 1 mm, and the discs formed by the sealers must be uniformly circular.

### Dimensional change

For the dimensional change test, five Teflon molds were used to obtain cylindrical specimens measuring 12 mm in diameter and 6 mm in thickness. These molds were placed on a glass plate covered with cellophane film. After mixing the sealers, the molds were filled until a slight excess was visible at the upper edge. Then, microscope glass slides were pressed against the open ends of the molds, and the assembly was secured using a C-shaped clamp.

After 5 min from the start of mixing, the assembly was transferred to an incubator set at 95% relative humidity and 37 °C, where it was kept for a period equivalent to three times the sealer’s setting time. Subsequently, the assembly was removed from the chamber, and the specimen surfaces were leveled using 600-grit sandpaper under constant irrigation with deionized distilled water.

Afterward, the specimens were removed from the molds, and their lengths were measured with a digital caliper. Once the measurements were taken, the specimens were placed in 10 mL containers filled with 2.24 mL of deionized distilled water at 37 °C and stored in an incubator for 30 days.

Finally, after the predetermined period, the samples were removed from the containers, dried with high-absorbency paper, and their lengths were measured again. The percentage of dimensional change was calculated using the following mathematical formula:

$$\:\left[\right({C}_{after\:30\:days}-\:C)\:/\:C]\:\times\:\:100$$

In this formula, “C” refers to the initial length, and “C₃₀ days” represents the length after the experimental conditions described.

### Solubility

For the solubility test, ten circular Teflon molds measuring 7.75 mm in internal diameter and 1.5 mm in thickness were secured with utility wax onto glass plates lined with cellophane and filled with the endodontic cement, which was in a softened consistency. An impermeable nylon thread was placed over each mold, and another glass plate covered with cellophane was manually pressed on top of each mold so that the plates made uniform contact with the mold surfaces. The entire assembly was maintained in an environment at 37 °C and 95% relative humidity for a period equivalent to three times the setting time. After this period, each specimen was weighed three times using a precision balance (MLW).

Subsequently, the samples were suspended by the nylon thread and placed in containers containing 7.5 mL of deionized distilled water, ensuring that the specimens did not come into contact with the inner walls of the containers. The assembly was kept in an incubator at 37 °C for seven days.

After this period, the samples were removed from the containers, rinsed with deionized distilled water, blotted dry with absorbent paper, and placed in a desiccator for 24 h before being reweighed. Following the desiccation period, the samples were weighed again. The mass loss of each sample was expressed as a percentage of the original mass, calculated according to the formula (original mass – final mass = % mass loss), and the resulting value represented the solubility of the cement. The measurements were rounded to the nearest 0.001 g.

### Radiopacity

For the radiopacity test, five acrylic plates (2.2 cm × 4.5 cm × 1 mm) were fabricated, each containing a perforation with an internal diameter of 5 mm. The perforations were filled with the cement and covered with a glass plate lined with cellophane paper, applying gentle pressure to remove excess material. After a period, equivalent to three times the setting time, the specimens were obtained.

The acrylic plates were positioned at a focus-object distance of 30 cm, and another acrylic plate (2 mm × 1.3 cm × 4.5 cm) was placed on top to stabilize a 99% aluminum step wedge (alloy 1100) with thicknesses ranging from 1 mm to 10 mm in uniform 1-mm increments. This setup allowed for comparison of all steps in a single radiographic exposure.

Radiographic images of the specimens and the step wedge were then acquired using an X-ray unit (Spectro 70X, Dabi Atlante, Ribeirão Preto, SP, Brazil) operated at 70 kVp, 8 mA, and an exposure time of 0.2 s. The imaging plates, after exposure, were scanned using the laser optical reader of the Digora digital radiographic system (Soredex Orion Corporation, Helsinki, Finland), which processed the images. The Digora for Windows 1.51 software provides, among other features, a radiographic density measurement tool (densitometric analysis), which determines the radiopacity of a given material based on its grayscale values. For standardization, an area of 2 mm² (44.5 × 44.5 pixels) was selected for each specimen in the radiographic images of the cements.

For the evaluation of the bond strength (BS) tests and the adhesive interface quality of the bioactive cements BioRoot Flow and BioRoot RCS, human tooth samples were used.

### Sample selection and preparation

After approval of the project by the Research Ethics Committee of the School of Dentistry of Ribeirão Preto, University of São Paulo, single-rooted human teeth with a minimum length of 15 mm (measured with a digital caliper; Digimess, Shiko Precision Ganging Ltd., China), from the cementoenamel junction to the root apex, were obtained from the Human Tooth Biobank of FORP-USP. The teeth were stored in 0.1% thymol solution at 9 °C until the beginning of the experiments.

The teeth were rinsed under running water for 24 h and subsequently cleaned using ultrasonic scaling (Profi II Ceramic, Dabi Atlante Ltda., Ribeirão Preto, SP, Brazil). Next, the teeth were examined macroscopically and radiographed (Spectro 70X Electronic, Dabi Atlante, Ribeirão Preto, SP, Brazil) in orthoradial and mesioradial directions using a digital sensor (Fona CDRelite, Schick, DMM, Bandeirantes, PR, Brazil) to select 30 teeth that met the following criteria: straight and fully formed root, single canal, absence of calcifications, resorptions, or cracks, and a buccolingual-to-mesiodistal dimension ratio ≤ 1.5. All endodontic procedures and root canal obturation procedures were performed by a single experienced operator in order to standardize the experimental procedures.

The selected teeth were coronally sectioned near the dentin-enamel junction using a double-faced diamond disk (KG Sorensen, Barueri, SP, Brazil) at low speed under irrigation to standardize root length to 15 mm, using an Isomet 1000 cutting machine. Initially, the root canals were irrigated with 2.5% sodium hypochlorite (NaOCl) using a disposable plastic syringe (Ultradent Products Inc., South Jordan, UT, USA) and a NaviTip needle with a 0.3 mm diameter (Ultradent Products Inc., South Jordan, UT, USA). Canal exploration was performed with a stainless-steel K-file #15 (Dentsply Sirona Endodontics, Ballaigues, Switzerland) in a passive motion until the tip reached the apical foramen, establishing the actual tooth length. From this measurement, 1 mm was subtracted to determine the working length (WL).

The biomechanical preparation was performed using the reciprocating instrumentation technique with the Reciproc size 50 taper 0.05 instrument (VDW GmbH, Munich, Germany), coupled to a Sirona 6:1 reduction handpiece (SN 25185; VDW GmbH, Munich, Germany) attached to the SMR 114,058 micromotor (VDW GmbH, Munich, Germany), which was connected to the VDW Silver electric motor (VDW GmbH, Munich, Germany). The instrument was used in a passive “pecking” motion and, after every three in-and-out movements, it was withdrawn from the canal and cleaned with gauze until the working length (WL) was reached. After each removal, the root canal was irrigated with 2.5% sodium hypochlorite (NaOCl), followed by aspiration and reinundation using a disposable plastic syringe and NaviTip needle. This was followed by final irrigation with 2 mL of 17% ethylenediaminetetraacetic acid (EDTA) for 5 min, irrigation with 2 mL of 2.5% NaOCl, and a final rinse with 20 mL of saline solution to neutralize the irrigating solutions, using a disposable syringe and NaviTip irrigation needle (Ultradent Products Inc., South Jordan, UT, USA). Canal drying was performed using White Mac suction cannulas and Capillary Tips (Ultradent Products Inc., South Jordan, UT, USA), followed by R50 absorbent paper points (Reciproc, VDW GmbH, Munich, Germany).

AH Plus was used as the reference material due to its well-established physicochemical properties and bond strength to root dentin, and it is widespread use as a benchmark (gold standard) sealer in endodontic research. BioRoot Flow and BioRoot RCS were compared with this material. Experimental conditions were established in accordance with standardized testing protocols described in ANSI/ADA Specification No. 57 and ISO 6876:2012. The sample size (*n* = 10 teeth per group) was based on previous studies evaluating push-out bond strength and physicochemical properties of endodontic sealers.

After instrumentation, the specimens were randomly divided into three experimental groups according to the pre-mixed bioceramic sealer used (*n* = 10): BioRoot Flow and BioRoot RCS. The fit of the R50 gutta-percha cone (Reciproc, VDW GmbH, Munich, Germany) at the WL was verified through digital radiographs taken in orthoradial and mesioradial directions, and obturation was performed using the single-cone technique.

The bioactive sealers BioRoot Flow and BioRoot RCS (Septodont, Saint-Maur-des-Fossés, France) were applied using the applicator syringe provided by the manufacturer. The syringe tip was positioned 3 mm short of the root apex, and the plunger was gently depressed to prevent excessive extrusion of the material. Subsequently, the previously selected gutta-percha cone, coated with the respective sealer, was inserted into the canal.

Excess filling material at the canal orifice was removed using a Paiva condenser (Golgran Indústria e Comércio de Instrumentos Odontológicos Ltda., São Caetano do Sul, SP, Brazil) previously heated in a flame. Then, while the gutta-percha remained plasticized, vertical compaction was performed with the cold Paiva condenser, applying light apical pressure for 5 s. Final cleaning of the canal entrance was carried out using a sponge moistened with alcohol, and the quality of the obturation was verified by orthoradial and mesioradial digital radiographs. The specimens were stored in numbered Eppendorf tubes and maintained at 37 °C and 95% relative humidity for a period equivalent to three times the setting time of the sealers.

### Tooth sectioning

For slice preparation, the samples were positioned on acrylic resin plates with their longitudinal axes parallel to the plate surfaces and fixed with hot glue, ensuring that the roots were sectioned perpendicularly to their long axes in the mesiodistal direction using a 0.3 mm-thick diamond disk (South Bay Technology, San Clement, CA, USA) under continuous water cooling, at a speed of 350 rpm and a load of 75 g, using an Isomet 1000 cutting machine.

From each root third, three dentin slices with a thickness of 1.0 mm (± 0.1 mm) were obtained. The first two slices from each third were reserved for the push-out bond strength test, followed by failure pattern analysis, totaling six slices per root. The most apical slice from each third was used for the evaluation of the adhesive interface quality under confocal laser scanning microscopy (CLSM).

### Push-out bond strength test

The slices were positioned on a stainless-steel metallic base attached to the lower portion of a universal testing machine (Instron 25-19−106; Instron, Canton, MA, USA), which contained a central orifice of 2.5 mm in diameter. Each specimen was aligned with the orifice, with its coronal surface facing downward. This methodology ensured precise and reproducible alignment during testing, allowing the plunger tip to be centered on the filling material and to avoid contact with the dentin during loading and displacement. Metallic plungers with active tip diameters of 1.0 mm, 0.6 mm, and 0.4 mm were used for the coronal, middle, and apical thirds, respectively. These plungers were attached to the upper portion of the testing machine and positioned directly over the intracanal filling material. The test was performed at a crosshead speed of 0.5 mm/min until maximum stress was reached.

The debonding force required for material displacement was recorded in Newtons (N). To calculate the bond strength, the force value was converted to Megapascals (MPa) by dividing it by the lateral bonded area (SL) of the intracanal filling material. To determine the bonded area, the geometric configuration of the filling material (endodontic sealer + gutta-percha) was considered based on the level of sectioning used to obtain the slices. Prior to testing, the height (h) of each slice was measured using a digital caliper, and the major and minor radii were obtained under a stereomicroscope (Leica M165C; Leica Microsystems, Wetzlar, Hesse, Germany) using the LAS v4.4 software (Leica Microsystems, Wetzlar, Hesse, Germany).

Thus, the lateral bonded area (SL) of the endodontic sealer (in mm²) was calculated using the following formula:

$$\:SL=\:(R\hspace{0.17em}+\hspace{0.17em}r)\:h2+(R-r)2$$

In this formula, “R**”** represents the radius of the filling material at its coronal portion, “r” represents the radius at its apical portion, and “h” is the height/thickness of the slice. Based on these measurements, the bond strength (BS), expressed in MPa, was calculated by dividing the force required to displace the gutta-percha (F) by its lateral bonded area (SL**)**, according to the equation: (RU = F/SL).

### Failure pattern analysis

After the bond strength test, the slices were examined to determine the failure pattern using a stereomicroscope (Leica M165C, Leica Microsystems, Wetzlar, Germany) at 10× to 25× magnification. The failures were quantified in percentages and classified as follows: (a) Adhesive to dentin: when the filling material detached from the dentin surface; (b) Adhesive to the filling material: when the gutta-percha detached from the endodontic sealer; (c) Mixed adhesive: when the gutta-percha detached from both the dentin and the endodontic sealer; (d) Cohesive in dentin: when fracture occurred within the dentin; (e) Cohesive in the filling material: when fracture occurred within the filling material itself.

### Adhesive interface analysis by confocal laser scanning microscopy (CLSM)

The third slice from each root third, totaling three slices per specimen (*n* = 10), was subjected to a qualitative and quantitative evaluation of the adhesive interface using confocal laser scanning microscopy (CLSM). The qualitative analysis of the adhesive interface was conducted by a single calibrated operator. Prior to the analysis, the examiner underwent a calibration process using representative images not included in the final sample. The evaluations were repeated after a 2-week interval to ensure consistency of the qualitative assessment.

Initially, the slice surfaces were standardized using 600- and 1200-grit silicon carbide abrasive papers, each applied for 1 min, with the aid of a precision holder to ensure flatness. The slices were then polished using a polishing machine (APL-4, Arotec S/A Ind. e Comércio, São Paulo, SP, Brazil) with felt discs of 0.3 and 0.05 μm, together with alumina paste (Arotec S/A Ind. e Comércio, São Paulo, SP, Brazil). Finally, the surfaces were conditioned with 17% EDTA for 10 s, rinsed with distilled water, and gently dried with absorbent paper.

For analysis, the specimens were positioned parallel to the stage of a 3D confocal laser scanning microscope (LEXT OLS4000, Olympus Corporation, Japan) to obtain images of the dentin/filling material interface using the OLS4100 software. Representative images from each slice were captured with a 20× objective lens to assess the adaptation of the filling material. The ImageJ^®^ software was used to quantify voids or gaps, performing 12 equidistant measurements along the adhesive interface, from the canal wall to the most distant point of each gap.

According to the methodology described by Balguerie et al. (2011), the adaptation of the sealer to the root canal wall was classified based on the following criteria:

- Good: most sections show no gaps between sealer and dentin
- Fair: most sections show a few small defects (< 1 μm) between sealer and dentin
- Poor: most sections show several gaps (1–10 μm) between sealer and dentin
- No adaptation: most sections show no sealer adherence to dentin (gaps > 10 μm).

### Statistical analysis

The bond strength data did not meet the normality assumption according to the Shapiro–Wilk test (*p* < 0.050). Nevertheless, a two-way ANOVA was applied, considering the lack of an appropriate nonparametric equivalent for this statistical model [36]. For the remaining quantitative variables with normal distribution (physicochemical properties), a one-way ANOVA was performed, followed by Tukey’s post hoc test.

The association between the failure pattern and the root canal sealer was analyzed using the Chi-square test, while the adaptation of the sealer to dentin was evaluated through the Kruskal–Wallis test, followed by the Dwass–Steel–Critchlow–Fligner post hoc test.

All analyses were performed using Jamovi software version 2.6 (The Jamovi Project, Sydney, NSW, Australia), adopting a 5% significance level.

## Results

Table 2 presents the results obtained from the analysis of the physicochemical properties of the evaluated endodontic sealers.

**Table 2:** Mean values and standard deviations of setting time (minutes), flow (mm), dimensional change (%), solubility (%), and radiopacity (mmAl) for BioRoot Flow, BioRoot RCS, and AH Plus endodontic sealers

Properties	Sealers		
	AH Plus	BioRoot Flow	BioRoot RCS
Setting Time (min.)	480 ± 2,78 ^C^	2007 ± 0,34 ^A^	570 ± 2,54 ^B^
Flow (mm)	53,2 ± 0,46 ^A^	26,4 ± 0,34 ^B^	25,1 ± 0,2 ^C^
Dimensional Change (mm)	0,006 ± 0,015 ^A^	0,042 ± 0,1 ^A^	0,012 ± 0,03 ^A^
Solubility (%)	0,52 ± 0,04 ^B^	9,68 ± 5,08 ^A^	6,15 ± 3,92 ^AB^
Radiopacity (mmAl)	17,22 ± 0,26 ^A^	3,24 ± 0,28 ^C^	8,34 ± 0,38 ^B^

*Different uppercase letters indicate statistically significant differences between the sealers (columns)

### Setting time

The AH Plus endodontic sealer exhibited the lowest setting time values, showing a statistically significant difference when compared with BioRoot Flow (*p* < 0.001), which presented the highest setting time values, and BioRoot RCS (*p* = 0.021), which showed intermediate values. Only AH Plus met the ANSI/ADA Specification No. 57, which stipulates that the deviation from the setting time stated by the manufacture should not exceed 10%.

### Flow

The mean flow values were as follows: AH Plus (53.2 ± 0.46 mm), BioRoot Flow (26.4 ± 0.34 mm), and BioRoot RCS (25.1 ± 0.2 mm) (*p* < 0.05). All sealers met the ISO 6872:2012 standard (minimum of 17 mm). AH Plus showed the highest flow value, followed by BioRoot Flow and BioRoot RCS.

### Dimensional change

Dimensional changes were as follows: AH Plus (0.006 ± 0.0015%), BioRoot Flow (0.042 ± 0.1%), and BioRoot RCS (0.012 ± 0.03%), with no statistically significant differences (*p* = 0.492). All evaluated sealers complied with the standards, remaining within the established limits (≤ 1% expansion or contraction).

### Solubility

BioRoot RCS exhibited behavior similar at times to BioRoot Flow (*p* = 0.323), which showed the highest values, and at other times to AH Plus (*p* = 0.080). Only AH Plus met the criteria established by ANSI/ADA Specification No. 57, which stipulates that endodontic sealers must not exceed a 3% mass change.

### Radiopacity

Radiopacity was highest for AH Plus, intermediate for BioRoot RCS, and lowest for BioRoot Flow, with a statistically significant difference (*p* < 0.001). All evaluated sealers met the minimum criterion of 3 mm Al established by the ANSI/ADA Specification.

### Push-out shear bond strength analysis

The push-out bond strength results (MPa) of the experimental groups are presented in Table 3.

**Table 3:** Mean push-out bond strength values and standard deviations (Mpa) for BioRoot Flow, BioRoot RCS and AH Plus sealers in coronal, middle and apical thirds of root dentin

	Sealers		
	AH Plus	BioRoot Flow	BioRoot RCS
Cervical	4,10 ± 1,64 ^Aa^	2,69 ± 0,96 ^Aab^	1,78 ± 1,15 ^Ab^
Middle	3,19 ± 2,19 ^Aa^	2,38 ± 1,37 ^Aab^	1, 19 ± 1,00 ^Ab^
Apical	2,43 ± 1,37 ^Ba^	2,10 ± 1,39 ^Aa^	0,80 ± 0,51^Aa^

* Different uppercase letters indicate differences between rows (root thirds). Different lowercase letters indicate differences between columns (sealers) (*p* < 0.05)

The Two-Way ANOVA test revealed statistically significant differences for the factors “root third” (*p* = 0.035) and “sealer” (*p* < 0.001). Tukey’s post hoc test showed that, in the AH Plus group, the cervical third exhibited higher bond strength values compared with the apical third (*p* = 0.037), while the middle third demonstrated intermediate behavior, being similar either to the apical (*p* = 0.857) or to the cervical third (*p* = 0.651). For the bioceramic sealers, no significant differences were observed among the root thirds (*p* > 0.05).

When comparing the sealers, in the cervical third, AH Plus exhibited higher values than BioRoot RCS (*p* < 0.05). BioRoot Flow demonstrated intermediate behavior, being sometimes similar to AH Plus (*p* = 0.67) and sometimes to BioRoot RCS (*p* = 0.525). A similar pattern was observed in the middle third, where AH Plus presented higher values than BioRoot RCS (*p* < 0.001), while BioRoot Flow again showed intermediate behavior, being similar either to AH Plus (*p* = 0.692) or to BioRoot RCS (*p* = 0.184). In the apical third, no statistically significant differences were found among the sealers.

### Failure pattern analysis

The failure pattern data for the different root canal sealers, expressed as percentages, are presented in Table 4.

**Table 4:** Failure mode after push-out test for the evaluated root canal sealers

Failure Type(%)	Sealer		
	AH Plus	Bio Root Flow	Bio Root RCS
AMO	56,6	21,1	37,3
M	43,4	78,9	62,7

*Failure type: AMO= Adhesive of obturation material; M= Mixed

The chi-square test revealed a statistically significant association between the type of failure and the variable “sealer” (*p* = 0.001). In contrast, no significant difference was found for the factor “root third” (*p* = 0.509). Accordingly, the test indicated a higher prevalence of mixed adhesive failures for the bioceramic sealers, whereas the AH Plus sealer predominantly exhibited adhesive failures at the sealer interface.

#### Adhesive interface analysis by confocal laser microscopy (CLSM)

Table 5 presents the results obtained from the analysis of the adhesive interface using CLSM. The Kruskal–Wallis test revealed no statistically significant difference for the factor “root third” (*p* = 0.773), indicating that the adaptation of the adhesive interface was similar in the cervical, middle, and apical thirds. However, when considering the different root canal sealers, the Kruskal–Wallis test showed a statistically significant difference (*p* < 0.001). The Dwass–Steel–Critchlow–Fligner post hoc test demonstrated that AH Plus exhibited significantly superior adaptation compared to BioRoot Flow (*p* = 0.002) and BioRoot RCS (*p* < 0.001), with no significant difference observed between the two bioceramic sealers (*p* = 0.491).

**Table 5:** Percentage distribution of gap-free, partial gap and complete gap patterns at the sealer-dentin interface for BioRoot Flow, BioRoot RCS and AH Plus in coronal, middle and apical thirds as assesed by confocal laser scanning microscopy (CLMS)

Score	Sealers		
	AH Plus	Bio Root Flow	Bio Root RCS
Good	21,7	0	0
Fair	78,3	61,5	75,9
Poor	0	38,5	24,1
NA	0	0	0

*Adaptation criterion of the sealer to root dentin: Good, Fair, Poor, No Adaptation, Abbreviation: NA – noadaptation

**Fig. 1:**
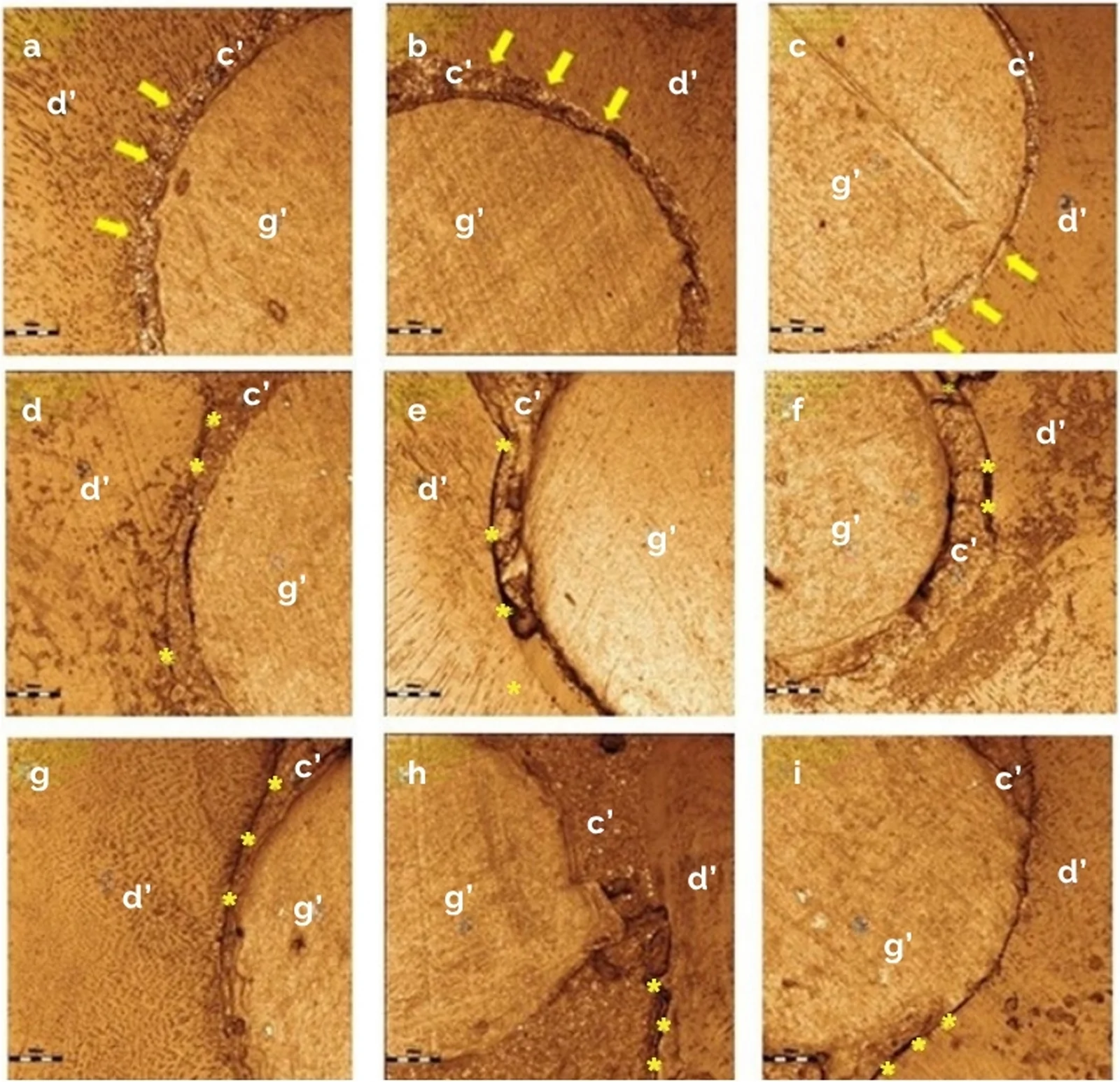
Confocal laser scanning microscopy photomicrographs (20× magnification) of the adhesive interface of the filling materials AH Plus, BioRoot Flow, and BioRoot RCS in the cervical (a, d, and g), middle (b, e, and h), and apical (c, f, and i) thirds. In white, the gutta-percha (g’), sealer (c’), and dentin (d’) are indicated, while the yellow markings highlight regions of juxtaposition (→) and gaps (*). The scale bar shown in the images corresponds to 100 μm

Qualitative analysis of the CLSM images revealed superior adaptation of the AH Plus sealer, with areas of close contact along the adhesive interface, indicated by yellow arrows (Fig. 1a and b, and 1c). In contrast, the images of the BioRoot Flow and BioRoot RCS sealers showed regions of interfacial gaps between the sealer and the dentinal wall, indicated by yellow asterisks (Fig. 1d, e, f, g and h, and 1i).

## Discussion

The search for obturation materials with suitable physicochemical properties and predictable clinical performance has been a continuous focus of investigation in Endodontics. Calcium silicate–based sealers have emerged as a promising alternative to other types of endodontic sealers, primarily due to their potential biocompatibility, antimicrobial activity, and favorable biological properties [11]. However, the introduction of new materials and formulations to the market—such as BioRoot Flow, a ready-to-use paste—requires a thorough evaluation of their compliance with international standard specifications.

Considering that the performance of an endodontic sealer directly impacts the success of root canal treatment, the evaluation of parameters such as setting time, flow, dimensional change, solubility, radiopacity, and bond strength to root dentin is essential. The literature emphasizes that no single material evaluated to date fully meets the requirements established by ANSI/ADA Specification No. 57, as each presents its own advantages and limitations [15–17]. Although bioceramic sealers are biologically promising, they still exhibit certain physicochemical limitations when compared with the epoxy resin–based sealer AH Plus. Previous studies have highlighted the challenge of achieving a balance between adequate physicochemical properties and bioactivity in the formulation of new endodontic sealers [15, 37, 38]. Furthermore, several investigations have demonstrated that the bond strength of calcium silicate–based sealers to dentin is lower than that of AH Plus, which remains the gold standard among root canal sealers [20, 26, 39–44].

Regarding the results, the literature indicates that the setting time of endodontic sealers is influenced by their constituent components, particle size, ambient temperature, and relative humidity [10, 33]. A significant difference was observed among the evaluated sealers, with BioRoot Flow showing the longest setting time, BioRoot RCS an intermediate one, and AH Plus the shortest. These findings are consistent with previous studies reporting that the prolonged setting of bioceramic sealers is related to their hydration-dependent setting reaction, which varies according to local moisture availability [9, 10]. In this reaction, calcium silicates interact with water to form calcium silicate hydrate and calcium hydroxide [19, 45]. Specifically, for BioRoot Flow, its formulation includes propylene glycol (PG), which appears to directly interfere with the kinetics of the setting reaction. Studies have shown that the addition of PG to calcium silicate–based sealers increases the setting time, although it improves handling and flow characteristics [46, 47]. This prolonged setting time does not appear to be an isolated limitation of BioRoot Flow, but rather a systematic characteristic of premixed (“ready-to-use”) bioceramic formulations containing PG or similar organic vehicles [9, 10]. Comparative studies evaluating multiple premixed calcium silicate–based sealers have demonstrated that this type of formulation consistently exhibits longer setting times than powder-liquid formulations [9, 48]. The prolonged setting time observed in premixed formulations has been attributed to the presence of organic vehicles, such as PG, polyethylene glycol, or other water-soluble polymers, which are necessary to maintain the material in paste form and prevent premature hydration during storage [9]. In contrast, AH Plus exhibits a shorter setting time because its polymerization occurs through a chemical reaction between epoxy and amine groups, resulting in a relatively fast hardening process.

Flow is an important property related to the three-dimensional filling of the root canal system, as well as the ability of the sealer to fill irregularities and penetrate into dentinal tubules [28]. However, it should be noted that increasing a sealer’s flowability also raises the risk of extrusion beyond the apical foramen. Therefore, highly flowable endodontic sealers should be more biocompatible with periapical tissues and, ideally, bioactive [10]. According to the present results, all tested sealers met the minimum flow requirements established by ISO 6876. AH Plus exhibited the highest flowability, followed by BioRoot Flow and BioRoot RCS. The superior flow values observed for AH Plus may be attributed to its resin-based components—such as dibenzylamine, aminoadamantane, tricyclodecane diamine, and silicone oil—which contribute to lower viscosity [49, 50]. Some authors have emphasized that strict compliance with ISO standards for flow may not be essential for bioactive sealers [51]. This consideration is based on their bioactivity; studies have demonstrated that the formation of a mineral infiltration zone allows, as the formation of a mineral infiltration zone allows the sealer to integrate with the dentin substrate through the precipitation of calcium phosphate and hydroxyapatite-like structures at the sealer-dentin interface [21, 52, 53]. This bioactive mechanism creates a chemical bond that differs fundamentally from the mechanical interlocking of resin-based sealers [54, 55]. Consequently, a thicker film layer may not compromise the sealing ability of root canal fillings performed with bioactive sealers, as the chemical integration compensates for reduced physical penetration [51].

Regarding dimensional change, no significant differences were observed among the evaluated sealers. This similarity, despite differences in solubility, suggests that both bioceramic and resin-based sealers are capable of maintaining volumetric stability after setting. These findings are consistent with previous studies emphasizing that minimal expansion or contraction is essential to prevent marginal leakage [17]. Such results may be explained by the fact that dimensional change results not only from the dissolution of components but also from water absorption and the formation of new hydration products [50, 56]. In calcium silicate–based sealers, the setting reaction generates calcium silicate hydrate and calcium hydroxide crystals, promoting slight volumetric expansion that can compensate for mass loss due to solubility [17, 50, 57, 58].

Solubility reflects mass loss when a material is immersed in water, and high values may compromise the three-dimensional sealing of the root canal system. Among the tested sealers, only AH Plus met ANSI/ADA specifications (≤ 3% mass loss). BioRoot Flow exhibited the highest solubility, followed by BioRoot RCS, while AH Plus showed the lowest values [59].

The solubility of endodontic sealers is fundamentally determined by their chemical composition and molecular structure, not by physical or microstructural characteristics [60–62]. Calcium silicate-based sealers exhibit higher solubility due to their hydrophilic chemical composition and ionic dissolution processes [9, 50]. These materials contain tricalcium silicate (Ca₃SiO₅) and dicalcium silicate (Ca₂SiO₄), which undergo continuous hydration reactions in aqueous environments, releasing calcium (Ca²⁺) and hydroxyl (OH⁻) ions—a chemical process resulting in measurable mass loss [50, 58, 63, 64]. The hydration products, primarily calcium silicate hydrate (C-S-H) and calcium hydroxide (Ca(OH)₂), are more susceptible to aqueous dissolution than dense polymeric structures [6, 9, 48, 50, 61, 63]. Hydrophilic nanoparticles in the formulation increase surface area for water interaction, intensifying ionic dissolution [50, 58, 61, 65]. In contrast, AH Plus exhibits low solubility due to its highly cross-linked epoxy structure. Polymerization between epoxy and amine groups creates a dense three-dimensional network of covalent bonds, forming a hydrophobic matrix resistant to water penetration [46, 47, 60, 66]. This polymeric structure minimizes component leaching and prevents ionic dissolution, resulting in practically null solubility (< 1%) [6, 50, 60, 64]. Molecular simulations confirm that epoxy polymers resist aqueous dissolution through low molecular polarity, high cross-linking density, and restricted water diffusion [52, 58, 66]. Microstructural alterations observed after dissolution of calcium silicate-based sealers—such as pores, cracks, or voids—represent consequences of chemical dissolution, not primary causes [50, 58, 61, 62]. Spectroscopic analyses (EDS, FTIR, XRD) confirm that dissolution involves elemental chemical changes and ion release (Ca²⁺, OH⁻, silicate) that precede microstructural alterations [48, 50, 58, 64, 67]. The immersion medium influences solubility through specific chemical mechanisms. In phosphate-buffered solutions (PBS) or simulated body fluid (SBF), phosphate (PO₄³⁻) and calcium (Ca²⁺) ions promote precipitation of calcium phosphate and hydroxyapatite precursors on bioceramic sealer surfaces, reducing apparent solubility by up to 50% compared to distilled water [6, 9, 60, 61, 67, 68]. This difference reflects variations in available chemical reactions rather than merely ionic composition [6, 9, 60, 61].

The finding that BioRoot Flow exceeded the 3% ANSI/ADA solubility limit warrants critical discussion regarding its potential clinical implications. While elevated solubility raises theoretical concerns about long-term sealing stability, the clinical significance must be interpreted within the context of the material’s bioactive properties and the limitations of in vitro testing [48, 64]. Long-term studies on calcium silicate-based sealers suggest that initial solubility may not directly correlate with clinical failure. Jalan et al. (2026) demonstrated that while BioRoot RCS exhibited solubility exceeding ISO standards at 24 h, the material showed reduced dissolution over extended periods (6 months) in phosphate-buffered saline, suggesting stabilization over time [6, 9]. This temporal pattern indicates that early mass loss may represent surface dissolution and ion exchange rather than progressive material degradation [6, 9, 50, 61]. Futhermore, the solubility of bioceramic sealers is accompanied by the release of calcium and hydroxide ions, which promote alkalinization of the periapical environment and precipitation of hydroxyapatite-like structures at the material-dentin interface [6, 9, 10, 63]. This bioactive response may compensate for initial material loss by creating a mineral infiltration zone that enhances sealing through chemical integration rather than mechanical retention alone [52].

However, the clinical implications of exceeding the 3% threshold cannot be dismissed entirely. Elevated solubility may lead to gap formation at the sealer-dentin interface, potentially compromising the apical seal and creating pathways for bacterial microleakage [9, 26, 60, 66]. Komabayashi et al. (2020) noted in their comprehensive review that while calcium silicate sealers demonstrate favorable antimicrobial and biocompatibility properties, their higher solubility compared to epoxy resin sealers remains a concern that requires long-term clinical validation [69]. The critical question is whether the bioactive sealing mechanism of BioRoot Flow can adequately compensate for its higher solubility in clinical conditions over extended periods. Current evidence from short-term clinical studies has not demonstrated increased failure rates with bioceramic sealers despite their higher laboratory solubility, but long-term clinical data specifically on BioRoot Flow are lacking. Therefore, while the material’s bioactivity may mitigate concerns about solubility in many clinical scenarios, clinicians should exercise caution in cases requiring maximum sealing stability, such as teeth with large periapical lesions or compromised root structure, until more definitive long-term clinical evidence becomes available [9, 26, 60, 66].

It is important to emphasize that solubility tests performed in distilled water, as recommended by the ANSI/ADA standard, may not fully reflect the behavior of calcium silicate–based sealers when placed inside the root canal. The intracanal environment consists of dentinal fluid enriched with ions such as calcium, phosphate, and magnesium, which may influence the dissolution and bioactivity of these material’s [6, 9, 64, 70]. Studies have demonstrated that the solubility of bioceramic sealers is reduced when tested in phosphate-buffered solutions compared with distilled water, although it may still exceed the limits established by ISO standards [6, 9, 62, 63]. Therefore, the reported solubility values should be interpreted considering this methodological limitation [6, 9, 64, 70].

Radiopacity is an essential property for radiographic assessment of the quality of root canal obturation and is primarily influenced by the type and concentration of radiopacifying agents incorporated into the formulation [37, 50, 58]. It can therefore be inferred that the amount and proportion of each radiopacifier determine whether a sealer exhibits higher or lower radiopacity [58, 71]. This likely explains the higher radiopacity values observed for AH Plus, which contains zirconium oxide and calcium tungstate—both known to enhance radiopacity [28, 50, 72]. In contrast, the bioceramic sealers contain only zirconium oxide, and in lower concentrations, which may account for the lower radiopacity values obtained [50, 58]. According to ANSI/ADA Specification No. 57, endodontic sealers must exhibit a minimum radiopacity equivalent to 3 mm of aluminum. In this context, all sealers evaluated in the present study met the international standard requirements [9, 10, 50].

The bond strength of endodontic sealers to root canal walls is a critical parameter for the clinical success of obturation, as it directly influences marginal sealing, material stability, and the prevention of coronal and periapical leakage [73, 74]. The push-out shear bond strength test revealed significant differences among the evaluated materials. The group obturated with AH Plus exhibited the highest bond strength values, followed by BioRoot Flow and, finally, BioRoot RCS. This result can be attributed to the formation of covalent bonds between the epoxy resin and the amino groups of dentinal collagen [39–41], leading to a stronger adhesion of AH Plus to dentin compared with the interaction between calcium silicate–based sealers and the dentin structure [26, 44, 75]. Additionally, for AH Plus, bond strength varied according to the root region—being higher in the cervical and middle thirds and lower in the apical third. This pattern may be explained by the greater number and diameter of dentinal tubules, as well as the larger amount of intertubular dentin in the cervical third [76–78]. In contrast, the apical third presents reduced dentin permeability and more limited technical access, which hinders sealer penetration and interaction, thereby accounting for the lower values observed [79, 80]. Furthermore, MCSL analysis revealed that AH Plus provided significantly better adaptation at the adhesive interface compared with the bioceramic sealers.

BioRoot Flow exhibited an intermediate performance compared with the other evaluated sealers and showed no significant differences among the root thirds, displaying bond strength values similar to AH Plus in the apical and middle regions. This behavior may be attributed to its calcium silicate–based bioceramic formulation, which promotes bioactivity, apatite formation, and the establishment of a chemical seal at the sealer–dentin interface [26, 43, 81, 82]. Recent studies support this hypothesis, demonstrating that BioRoot Flow possesses favorable biological properties, including high cytocompatibility, induction of cell differentiation, and the formation of mineralized nodules [6, 10, 71, 83]. In addition, its formulation ensures continuous release of calcium and hydroxide ions even under adverse conditions, such as thermal stress, promoting stable chemical interactions with dentin [6, 9, 10, 41]. Further evidence also indicates that pre-mixed bioceramic sealers, such as BioRoot Flow, exhibit consistent and uniform tubular penetration, which may explain their stable performance even in regions with lower tubular density, such as the apical third [75, 80, 84].

The inferior performance of BioRoot RCS compared with BioRoot Flow may be associated with its powder–liquid presentation and the resulting physicochemical characteristics derived from manual manipulation. Although both are calcium silicate–based bioceramic sealers, BioRoot RCS relies on the proportion and homogeneity of the mix to ensure proper setting reaction and apatite layer formation [26, 70, 82, 85]. Variations in the liquid amount or mixing technique can lead to porosity, increased solubility, and reduced internal cohesion, compromising the integrity of the adhesive interface [26, 41, 43, 85, 86]. Furthermore, studies indicate that BioRoot RCS exhibits lower flowability and reduced tubular penetration compared with pre-mixed formulations, which limits the extent of mineral infiltration zones and decreases bond strength, particularly in regions of lower dentin permeability such as the apical third [26, 37, 40, 43, 85]. These factors may explain the lower push-out bond strength values observed for BioRoot RCS

The literature highlights the challenge of controlling moisture within the root canal during obturation to ensure an ideal amount of water capable of allowing proper material setting and achieving suitable physicochemical properties and bond strength [87, 88]. In the present study, the canals were standardized as dry, following the manufacturers recommendations, in order to minimize variables and ensure result reproducibility. However, it is important to emphasize the distinction between the addition of 1% distilled water during the manipulation of BioRoot Flow and BioRoot RCS sealers to initiate their hydration reaction, and the moisture condition of the root canal walls during obturation, as previously reported. Although calcium silicate–based sealers are hydraulic materials, complete canal drying does not prevent adequate setting, since residual moisture within the dentinal tubules is sufficient to support the hydration reaction [9, 10]. Nevertheless, considering the findings of Pelozo et al. (2023), which reported higher bond strength values for pre-mixed bioceramic sealers when the canal exhibited residual moisture, whereas AH Plus showed superior bond strength under complete drying conditions, the standardized dry protocol employed in the present study may have favored AH Plus performance while potentially limiting the bond strength of BioRoot Flow and BioRoot RCS. Future investigations evaluating sealer-specific drying protocols may reveal higher bond strength values for the calcium silicate–based sealers tested.

Regarding the methodology used in the present study, the tests for the analysis of physicochemical properties followed the criteria established by ANSI/ADA Specification No. 57, as modified by ISO 6876:2012. To reduce the amount of endodontic sealer used in the tests, molds with reduced dimensions were employed, as proposed by Carvalho-Junior et al. (2007), who demonstrated that reducing the material volume by approximately 80% for specimen preparation does not affect the accuracy of the methods or the reliability of the results obtained.

The bond strength was evaluated using the push-out shear bond strength test, which allows assessment across different thirds of the root canal [87]. Bond strength is an essential parameter for assessing the adhesion of the root canal sealer to the dentinal walls, measuring its ability to resist dislodgement [26, 42, 43]. Clinically, this parameter is relevant because it reflects the long-term stability of the obturation, indicating the efficiency of apical sealing and reducing the risk of bacterial microleakage. For this purpose, metallic rods and bases with active tips and orifices of diameters compatible with each tested third were used, enabling the application of force and the distribution of shear stresses as close as possible to the adhesive interface between the sealer and the root dentin [73].

It is noteworthy that single-rooted teeth with a minimum root length of 15 mm were carefully selected. Furthermore, anatomical standardization using samples with circular root canal cross-sections allowed the formation of anatomically balanced experimental groups, thereby reducing the risk of bias in the results. Additionally, the analysis of the failure pattern after testing, performed under a stereomicroscope, complemented the interpretation of the bond strength data obtained.

The adhesive interface formed between the gutta-percha, sealer, and intraradicular dentin was analyzed using confocal laser microscopy (CLSM) images. To evaluate the adaptation of the sealers through the measurement of gaps and voids, the adhesive interface was divided into quadrants, classified, and statistically analyzed using scores.

The results of this study suggest that the superior performance of AH Plus in terms of bond strength, interfacial adaptation, and physicochemical properties reinforces its effectiveness in promoting sealing and obturation stability, particularly in the cervical and middle thirds. On the other hand, BioRoot Flow may represent an interesting alternative in clinical situations where bioactivity is a primary factor, supporting the indication of calcium silicate–based sealers.

The failure mode analysis provides critical insights into the underlying adhesion mechanisms. AH Plus predominantly exhibited adhesive failures at the sealer-dentin interface, characterized by clean separation from dentin without cohesive fracture of the sealer material itself [26, 43, 89]. This pattern indicates that the weakest point is the adhesive interface rather than the sealer material, reflecting that the epoxy resin’s internal cohesive strength exceeds the strength of its bond to dentin [26, 43, 89]. The prevalence of adhesive failures confirms that fracture occurs at the covalent bond interface between the epoxy resin and dentinal collagen, representing the mechanical limit of this chemical interaction [26, 43, 89].

In contrast, the bioceramic sealers (BioRoot Flow and BioRoot RCS) predominantly exhibited mixed adhesive failures, indicating that fracture occurred partially at the sealer-dentin interface and partially within the sealer material itself [26, 43]. This pattern reveals fundamentally different mechanical behavior and suggests that while the mineral infiltration zone provides interfacial adhesion through hydroxyapatite precipitation and chemical bonding to dentin, the cohesive strength of the sealer material may be a limiting factor.

Importantly, the occurrence of cohesive failures within bioceramic sealers suggests that the mineral infiltration zone—hypothesized to form through precipitation of hydroxyapatite-like structures at the sealer-dentin interface-may actually achieve stronger adhesion to dentin than the internal cohesive strength of the sealer itself [26, 43, 54, 55, 67, 75, 90]. This is a critical distinction: while AH Plus fails at the interface (adhesive failure), bioceramic sealers fail partially within the material (mixed failure), which is consistent with the hypothesis that the bioactive mineral deposition creates a robust chemical seal that integrates with the dentin substrate [26, 43, 54, 55, 67, 75, 90].

Although CLSM analysis evaluated interfacial adaptation and sealer penetration into dentinal tubules, it did not directly confirm mineral infiltration zone formation, as fluorescent dyes visualize sealer penetration but not hydroxyapatite precipitation [43, 54]. However, previous studies using SEM/EDS, FTIR, and XRD have demonstrated that calcium silicate-based sealers release calcium and hydroxyl ions that promote apatite formation at the sealer-dentin interface, creating a mineral-rich zone with immature crystallites exhibiting high remineralizing activity [6, 9, 52, 53, 91, 92]. Therefore, the mixed failure pattern observed in bioceramic sealers should not be interpreted as inferior adhesion, but rather as evidence of a different adhesion mechanism relying on mineral deposition and chemical integration with dentin rather than covalent bonding to collagen [26, 43, 75]. This bioactive mechanism provides excellent biocompatibility and promotes tissue healing, though it may result in lower immediate bond strength compared to epoxy resins [6, 10, 26, 52, 75].

The findings demonstrate that AH Plus exhibited superior bond strength, interfacial adaptation, and physicochemical properties through its dual adhesion mechanism—covalent bonding to dentinal collagen and mechanical interlocking via tubular penetration—accounting for its high bond strength and predominantly adhesive failure pattern [26, 43, 75]. BioRoot Flow demonstrated intermediate performance with stable bond strength across all root thirds, attributed to its calcium silicate formulation that promotes bioactivity and hydroxyapatite formation at the sealer-dentin interface [26, 43, 55]. Therefore, BioRoot Flow may represent a clinically viable alternative when bioactivity, biocompatibility, and tissue healing are prioritized, supporting the indication of calcium silicate-based sealers in specific clinical scenarios.

## Conclusion

Based on the results obtained in the present study, none of the evaluated sealers fully met the requirements established by ANSI/ADA Specification No. 57 for root canal sealers. The epoxy resin–based sealer AH Plus demonstrated the best overall performance, showing the lowest solubility values, high radiopacity, superior flow capacity, and greater bond strength to root dentin, particularly in the cervical and middle thirds. Among the evaluated calcium silicate–based sealers, BioRoot Flow exhibited higher bond strength values compared to BioRoot RCS.

Thus, although AH Plus remains the reference material in terms of physicochemical and adhesive properties, BioRoot Flow stands out as a viable alternative among bioceramic sealers, reinforcing the need for further studies aimed at improving formulations and gaining a deeper understanding of their long-term clinical behavior.

## Data Availability

No datasets were generated or analysed during the current study.
